# The Clinical Value of Nutritional Care before and during Active Cancer Treatment

**DOI:** 10.3390/nu13041196

**Published:** 2021-04-05

**Authors:** Giuseppe Aprile, Debora Basile, Renato Giaretta, Gessica Schiavo, Nicla La Verde, Ettore Corradi, Taira Monge, Francesco Agustoni, Silvia Stragliotto

**Affiliations:** 1 Department of Oncology, AULSS8 Berica, 36100 Vicenza, Italy; debora.basile@aulss8.veneto.it (D.B.); renatogiaretta1956@gmail.com (R.G.); 2 Clinical Nutritional Unit, AULSS8 Berica, 36100 Vicenza, Italy; gessica.schiavo@aulss8.veneto.it; 3 Department of Oncology, PO Sacco, ASST Fatebenefratelli Sacco, 20131 Milano, Italy; nicla.laverde@asst-fbf-sacco.it; 4 Clinical Nutritional Unit, ASST Grande Ospedale Metropolitano Niguarda, 20162 Milano, Italy; ettore.corradi@ospedaleniguarda.it; 5 Clinical Nutrition, S. Giovanni Battista Hospital, 10126 Torino, Italy; tairamonge@gmail.com; 6 Medical Oncology Unit, Fondazione IRCCS Policlinico San Matteo, 27100 Pavia, Italy; f.agustoni@smatteo.pv.it; 7 Department of Oncology, Istituto Oncologico Veneto—IRCCS, 31033 Padova, Italy; silvia.stragliotto@iov.veneto.it

**Keywords:** muscle wasting, malnutrition, nutritional intervention, nutritional care, sarcopenia

## Abstract

Malnutrition and muscle wasting are frequently reported in cancer patients, either linked to the tumor itself or caused by oncologic therapies. Understanding the value of nutritional care during cancer treatment remains crucial. In fact, cancer-associated sarcopenia plays a key role in determining higher rates of morbidity, mortality, treatment-induced toxicities, prolonged hospitalizations and reduced adherence to anticancer treatment, worsening quality of life and survival. Planning baseline screening to intercept nutritional troubles earlier, organizing timely reassessments, and providing adequate counselling and dietary support, healthcare professional may positively interfere with this process and improve patients’ overall outcomes during the whole disease course. Several screening tools have been proposed for this purpose. Nutritional Risk Screening (NRS), Mini Nutritional Assessment (MNA), Patient Generated Subjective Global Assessment (PG-SGA) are the most common studied. Interestingly, second-level tools including skeletal muscle index (SMI) and bioelectric impedance analysis (BIA) provide a more precise assessment of body composition, even if they are more complex. However, nutritional assessment is not currently used in clinical practice and procedures must be standardized in order to improve the efficacy of standard chemotherapy, targeted agents or even checkpoint inhibitors that is potentially linked with the patients’ nutritional status. In the present review, we will discuss about malnutrition and the importance of an early nutritional assessment during chemotherapy and treatment with novel checkpoint inhibitors, in order to prevent treatment-induced toxicities and to improve survival outcomes.

## 1. Introduction

The clinical approach to patients with advanced cancer has markedly changed in the last decade, moving to a more person-centered therapeutic plan with strong patients’ empowerment and engagement in all the possible aspects of care. The introduction in the clinical practice of novel, highly active drugs—especially target therapies or immune modulators-has been associated with extended survival, increased prevalence of cancer patients on treatment, and higher healthcare costs. At the same time, the age at diagnosis of cancer patients is progressively increasing as the prevalence of overweight or obese subjects [1,2]. In this changing context, accurate and timely nutritional care during active treatment remains crucial. In fact, in Italy and in other Countries not only malnutrition affects approximately half of cancer patients, but also it has important influences on their outcomes, since its effect results in negative consequences on outcomes [3,4]. Consequently, in addition to anticancer treatments, nutritional care plays a central role in the whole management of cancer patients. More especially, muscle wasting, resulting from mechanical and functional disorders including the imbalance between catabolic and anabolic pathways, is associated with increased surgical complications, poor prognosis, greater treatment-related toxicities, a poorer response to anti-cancer therapies, worse quality of life and length hospital stay [3,5,6].

However, despite the knowledge that an early intervention could influence clinical evolution of oncological processes and several tools currently available, a significant number of malnourished patients still remain undetected [7]. Of note, recent studies reported that only 30–70% of patients with risk of malnutrition received a nutritional assessment and half of them received an appropriate intervention [8,9,10,11]. In this short review, we discuss on the role of detecting malnutrition early, and how prevent this condition actively throughout the oncological care path. In particular, we focus on the importance of nutritional care during chemotherapy and treatment with novel checkpoint inhibitors, both as a measure to let more patients receive efficacious therapies and to prevent treatment-induced emergent side effects.

## 2. Detecting Malnutrition from the Very Beginning of Cancer Care: Who Starts Well Is Half the Battle

Since many cancer patients suffer weight loss and are poorly nourished or initially sarcopenic, the importance of screening patients for malnutrition from the beginning of their treatment is well established, as lack of proper nutritional management may limit the response to even the most effective therapy [12,13]. In cancer, the prevalence of malnutrition is one of the highest of all groups of disease, and the degree of weight loss depends on primary tumor location, type and extent of metastases, stage of the disease [14]. Unfortunately, nutritional assessment is provided only for the 30–60% of malnourished cancer patients in clinical practice [7], despite more than 50% of hospitalized cancer patients, and up to 30% of cancer outpatients are sarcopenic [15]. Indeed, a national survey conducted by the Italian Society of Medical Oncology (AIOM) and the Italian Society of Artificial Nutrition and Metabolism (SINPE), reports a nutritional assessment or dietary implementation for only 28% of cancer patients [16]. Likewise, despite the majority of specialists of Delphi panel believe that nutritional integration could improve quality of life, compliance and tolerability to chemotherapy, they perform a dietetic management in <30% of cancer patients on screening [17]. A questionnaire based survey administered to 357 UK Oncologists, report their high consideration about nutritional screening in cancer patients; however, patients at risk of malnutrition are not easily identified [18]. Interestingly, a Global Leadership Initiative on Malnutrition (GLIM) study compared in the world malnutrition prevalence, interventions and outcomes [19]. The GLIM consensus recommend to include both phenotypic and etiologic findings for the diagnosis of malnutrition to guide nutritional intervention and expected prognosis. However, only phenotypic criteria are recommended to define severity grading. The five criteria considered most important were: non-volitional weight loss, low BMI, muscle wasting, reduced food intake, disease inflammation [19]. 

Furthermore, the identification of muscle wasting may be potentially misleading since about 40–60% are overweight or obese; thus, obese patients require cautious baseline evaluation to determinate their nutritional risk [20]. Early screening to identify patients with sarcopenia and/or decreased muscle quality would allow earlier multimodal interventions to attenuate adverse body compositional changes [21]. Consequently, the purpose of any nutritional screening is to perform a baseline assessment of the nutritional status of the patients and to predict its worse or better clinical outcome depending on it [22]. 

Malnutrition associates with higher rates of morbidity, mortality, and treatment-related toxicities, with a negative prognostic power that potentially affects the overall treatment outcome [3,4,6,23,24]. Even in patients potentially cured, it is important to establish their nutritional risk, because not adequately receiving postsurgical therapies may hinder long term results. Patients who are unable to complete the adjuvant treatment due to malnutrition may lose the chance of cure compared to those who complete it, which may increase the chance of long-term survival of an absolute of 5 to 25%. 

The European Society for Clinical Nutrition and Metabolism (ESPEN) guidelines recognize the amount of weight loss—a number easy to determinate in every ward—as the most reliable indicator of nutritional deficit [12]. Moreover, ESPEN recommendations establish an individualized nutritional intervention focused on a multimodal approach [12]. However, in daily clinical practice, several questions still remain to be clarified, leading wide different manner to approach malnourished patients among countries. Therefore, the formulation of pragmatic guidelines to guide classification of risk’ patients and nutritional interventions represent an urgent unmet clinical need.

Basic objective anthropometric measures such as body mass index (BMI) and plicometry, in the last decade many strategies have been implemented in the daily clinical practice, to provide a more accurate but still simple measure for early detection and risk stratification of malnutrition [8,25,26]. Thereafter, patients at risk for malnutrition need preplanned evaluations through the therapeutic course and frequently require active intervention or regular follow-up. Although a universal agreement on screening methods is still lacking, classical baseline first-level tools recommended by ESPEN may include: (a) adult MUST (Malnutrition Universal Screening Tool), that better works for community outpatients where severe confounding factors are limited; (b) NRS-2002 (Nutritional Risk Screening 2002), which is more accurate in determining under-nutrition in hospital-based patients; (c) Mini Nutritional Assessment (MNA), which may be of greater help in the baseline assessment of senior cancer patients; and (d) PG-SGA (Patient Generated Subjective Global Assessment), which seems to have best diagnostic performance with cancer patients in a recent Bayesian comparison analysis [22]. 

Second-level tools entail specialist evaluations and tests that are more precise but more complex to administer and interpret. Recent studied have suggested that BMI measure may be biased and could provide inaccurate estimation of body composition and fat distribution because of its inadequacy to distinguish between muscle and adipose tissue [8,27]. Among novel techniques regarding biometric parameters, evaluations of computed tomography or magnetic resonance scans, cut at the level of the third lumbar vertebra, are emerging as promising and accurate approaches to provide a more precise assessment of body composition [28]. These evaluations require experienced radiologists and specific software that may measure cross-sectional areas of skeletal muscles and normalize them for height to obtain the skeletal muscle index (SMI). The prognostic value of these measures is very promising [28]. Bioelectric impedance analysis (BIA) is also a novel method, often used. A definitive, randomized comparison of the two methods has not been done, but exploratory comparisons suggest that image-based method is more accurate [29,30]. Although the Global Leadership Initiative on Malnutrition has recently proposed a consensus on the criteria to diagnose malnutrition both for inpatients and for outpatients [31], the worldwide validity of these criteria is still unconfirmed.

## 3. Nutritional Care during Active Chemotherapy: Any New Tricks for This Old Issue?

Unintentional weight loss, often combined with progressive skeletal muscle atrophy, is commonly reported during chemotherapy [32]. The pathogenesis of this pro-inflammatory phenomenon is multifactorial where prevention, early identification, and intervention remain important cornerstone in clinical practice. Many cytotoxic agents, given alone or more often in combination, commonly cause significant clinical toxicities which translate in pain, distress, reduced patients’ quality of life, and decreased treatment tolerance. The early stage of malnutrition, cancer cachexia, and sarcopenia are well-established risk factors for a scope of chemotherapy-induced toxicities, including individual symptoms such as fatigue, dysgeusia, mucositis, nausea/vomiting, hematologic toxicity, pain or composite clusters of these symptoms [33]. Patients with different cancers may be similarly affected, as indicate studies that included subjects with upper and lower gastrointestinal malignancies [23,34], breast cancers [35], lung tumors [36], or head and neck neoplasms [37,38]. Moreover, cancer malnutrition may be a significant risk factor for treatment-induced toxicities in more frail population, such as older patients, regardless their type of disease [39]. Malnutrition increase the chance of multiple side effects, and it may intensify their grade or prolong their duration. Induced either by the disease itself or by the treatments, sarcopenia may also affect the tolerance to chemotherapy and negatively influence the outcome of cancer patient. Among other toxicities, mucositis may affect any portion of the gastrointestinal tract. Its pathophysiology has been well described with a five-step process triggered by inflammatory cytokines that involves both the epithelium and the immune cells endowed in the submucosa [40]. Low body mass index, poor oral health and female sex have been identified as possible risk factors for oral mucositis. A number of possible methods to prevent, attenuate or cure this side-effect have been recently reviewed [41]. Taste and smell alterations may also be caused by chemotherapy regimens, particularly in previous smokers, females or in head and neck cancer patients, and may contribute to malnutrition [42,43]. Apart from epithelial damages, chemotherapy-induces changes in the expression of specific genes that have been linked to dysgeusia and to mild/moderate stomatitis [44].

The nutritional armamentarium in the management of weight-losing patients with cancer is based on counseling, non-pharmacological (nutrients or physical activity) and pharmacological supports [45,46].

Patients with risk of sarcopenia on screening, should be assessed for muscle mass, nutritional intake, physical performance, and the degree of systemic inflammation.

The total energy depletion of cancer patients should be individualized, usually, 25–30 kcal/kg per day, and protein intake should be >1 g/kg–1.5 g/kg per day to improve lean body mass and increase liver production of anabolic proteins [47]. 

Conversely, recommended carbohydrate intake is <5 g/kg per day. In particular, in sarcopenic subjects with insulin resistance, it is recommended to achieve most of the energy from fat compared to carbohydrates to reduce the glycemic load [12]. 

Interestingly, physical activity attenuates muscle wasting by increasing insulin sensitivity, suppressing inflammatory mediators and promoting protein synthesis [48,49].

About pharmacological approaches, in recent years, several potential agents have been studied such as zinc supplementations along with expert dietary counselling may improve the appetite and ameliorate clinical conditions of cancer patients being exposed to chemotherapy [50,51]. A proactive assessment of the nutritional status is essential for selecting the adequate intervention needed and it provides a reasonable cost-effective method to favorably impact on nutritional status, body composition, treatment tolerance and clinical outcome. Upcoming randomized clinical trials will clarify if treatment-naïve gastric cancer patients at risk for malnutrition might benefit from the addition of an early, short-term, supplemental parenteral nutrition to nutritional counseling alone [52].

Increasing evidence observed the key role played by active dietary interventions in cancer patient through oral or parenteral nutrition showing an improved energy intake and body weight. However, no impact has been reported on survival outcomes and treatment-related adverse events [53,54]. Intriguingly in head and neck cancer patients, nutritional support via ONS or enteral confers an increased adherence to anti-cancer treatments with reduced weight loss and hospitalization [55]. However, early detection of malnutrition and early management of nutritional status in parallel with oncological treatment could enhance anti-cancer therapies [56]. Dronabinol, the main psychoactive component of marijuana, has been tested to treat nausea and vomiting in patients not responding to conventional antiemetics and to increase appetite.

Although interesting on a theoretical basis, the possibility to starve cancer cells in order to increase the activity of chemotherapeutic agents is far unclear. There is a lack of evidence for suggesting fasting mimicking diets before oncological treatments in the clinical practice [57,58,59]. Prospective, well designed, randomized trials on caloric restriction or caloric restriction mimetics soon before or after chemotherapy to enhance treatment activity and decrease the rate and intensity of potential side effects are currently lacking [60]. Another critical point is the time for reassessing: how often should a cancer patient be reevaluated in order to check his nutritional status? Although the answer is not easy and may depend on specific disease, stage, and on patient’s prognosis, a monthly assessment could be suggested [61].

## 4. Nutritional Care during Immunotherapy: Does the Boundless Prairie Have a Fence?

Immunotherapy has recently emerged as a revolutionary novel standard of care in many tumors such as melanomas, lung cancer without genetic mutational drivers, deficient mismatch repair (dMMR) and microsatellite-high (MSI-H) gastrointestinal malignancies, triple negative breast cancers, and genitourinary malignancies. Immunomodulators may restore T-cell function and potentiate NK cells activity by blocking the ligand/receptors binding induced by cancer cells that cause T-cell inactivation. For a significant subset of cancer patients, immune checkpoint inhibitors (ICIs) produce durable, long-term responses. Nevertheless, since predictive factors are uncertain, it is not clear which patients may benefit the most from this new (and highly costly) treatment strategy. Several preclinical studies have suggested the possibility of an interplay between cell metabolism and susceptibility to immunotherapy. The contribution of neutrophils, monocytes, and T-cell types to myofiber composition and microenvironment has been documented, as well as to the processes of skeletal muscle damage, repair, remodeling, inflammation and fibrosis [62]. Along this line, a number of clinical studies have reported the association between BMI and the effectiveness of ICIs in patients with advanced melanoma [63], lung cancers [64], or triple negative breast tumors [65]. Despite this amount of data, results are still confounding. Baseline PNI (prognostic nutritional Index), for example, has been reported as useful in some retrospective studies [66], but questioned [67] or not confirmed in others [68]. In melanoma patients, overweight or obese patients may derive benefit in terms of progression-free survival (PFS), but not in overall survival (OS). If the issue may be limited to the evaluation of baseline, static data, instead of a more comprehensive dynamic nutritional pattern has been suggested [69,70]. 

The onset of anorexia/cachexia syndrome may also inhibit immunomodulators activity thus rendering the treatment less useful [71,72]. In a retrospective study of 156 non-small-cell lung cancer (NSCLC) patients treated with PD-1/PD-L1 inhibitors, those with high muscle quality showed higher response rate (35.0 vs. 15.8 %, *p* < 0.05) and longer PFS (median, 4.5 vs. 2.0 months, *p* < 0.05) than those with low muscle quality [73]. Another retrospective analysis of over 600 patients with advanced cancer who received immune checkpoint blockade, those with decreased pretreatment BMI and lower PNI had worse response rates (*p =* 0.0005), disease control rate (*p <* 0.0001) as well as shorter PFS (*p* = 0.02 and *p* = 0.02) and OS (*p* < 0.001) [69]. Despite higher BMI has been consistently associated with increased risk of treatment-related adverse events in patient receiving checkpoint inhibitors [74,75], in an Italian cohort analysis including 962 metastatic NSCLC patient treated with pembrolizumab and 426 treated with chemotherapy, baseline obesity correlated with significantly improved overall response rate (ORR), PFS and OS in patients with a PD-L1 expression of ≥50%, receiving first line pembrolizumab, but not among patients treated with chemotherapy [76]. The biological effect of adiposity on the host immune response triggered by checkpoint inhibitors is currently unclear [77], as it is not certain the underpinning background that sustains the opposite effect of visceral fat on the response to antiangiogenic drugs [78]. Moreover, cachectic patients may have an altered pembrolizumab clearance and pharmacokinetics (PK) values, which affect its action [79].

## 5. Nutritional Care during Later Treatment Lines and Beyond: Is the Final Hurdle Worth Jumping?

In many cancer types, it is quite common to offer patients with three or more treatment lines. While the need for supportive care increase with time (and with disease progression), the same may not be true for specific nutritional support. Although it is true that patients who become underweight need help to preserve lean body mass, to diminish nutrition-related side effects and to uphold their quality of life, to what extent nutritional care interventions, instead of comfort feeding, may increase the chance of achieving these goals is not cleared. A randomized prospective trial showed that home parenteral nutrition may have advantages, prolonging survival and improving quality of life in malnourished patients receiving home-based palliative care [80]. Other reports, however, indicate that the use of artificial nutrition, defined as a medical treatment that allows a non-oral mechanical feeding, in the end of life care is limited to 3% of the patients [81]. Despite this remains an unresolved issue [82,83], a tailored approach to optimize resources in every single patient is highly desirable.

## 6. Nutritional Care during Cancer Treatment: The Changing Role of Oncologists, Nutritionists and Nurses

The evidence that in the real practice malnutrition is often under recognized and undertreated in cancer patients [84] has promptly stimulated the blueprint of novel care pathways. In this scenario, to facilitate a renewed collaboration among healthcare professionals is crucial. Although to activate a formal nutritional team in every hospital setting may be not easy, some experiences have shown that this type of collaboration may be improved and, importantly, a shared awareness of this issue may benefit the patients’ care [85]. To enhance the cooperation of the teamwork and the collaboration with the patient community, many aspects should be enriched. Firstly, healthcare professionals involved in detecting patients at risk for malnutrition should share baseline nutritional screening tools to improve their confidence in advising cancer patients [86]. A recent survey showed that though oncologists, nurses and nutritionists discuss specific alimentary troubles with cancer patients and may provide information, many of them lack an awareness of guidelines and confidence in providing nutritional advice [87]. Secondly, adapted tools or apps may facilitate the engagement of patients within the nutritional track [88]; In Italy, we developed in collaboration with AIOM, SINPE and FAVO (Federazione Italiana delle Associazioni di Volontariato in Oncologia) the app Nutrient to easily screen patients at higher risk for malnutrition which may benefit from an early referral to experienced nutritionists [89]. Thirdly, simple interventions with the use of fact sheets [90] or educational videos [91] may improve knowledge and the overall understanding of malnutrition in cancer patients. In some hospitals there are procedures and operational methods that aim to guarantee the homogeneity of action of the healthcare professionals that a cancer patient may encounter. The use of validated and shared care pathways not only helps professionals to make adequate therapeutic decisions more quickly, but it ensures compliance with scientific recommendations, guarantees access to information, improves risk containment, rationalizes expenditures, and produces fairness.

## 7. Conclusions

Malnutrition is a multifactorial effect experienced by cancer-patients due to inflammation, imbalance between anabolic and catabolic pathways, anti-cancer toxicities, inadequate food intake and hormonal abnormalities. 

In the rapidly evolving cancer field, ranging from insights gained with the molecular dissection of the tumors to novel approved therapies, to assess frequently the nutritional status of our patients remains a critical aim regardless the disease stage. While during cancer treatments it is possible to experience reduction of daily oral intake, the efficacy of standard chemotherapy, targeted agents or even checkpoint inhibitors is potentially linked with the patients’ nutritional status. Literature evidence suggest that patients’ nutritional risk should be assessed early and monitored during the whole treatment course, with proactive measures, in order to improve tolerance, ameliorate quality of life, and achieve better clinical outcomes. To fight against malnutrition remains a common goal of all who make up the patients’ care team. Therefore, a close collaboration among experts and nutritional societies is necessary to promote pragmatic screening tools and guidelines to better define the timing of nutritional intervention in malnourished patients.

## Data Availability

No data are published in this article. This is a review article.
